# Migration of Chadic speaking pastoralists within Africa based on population structure of Chad Basin and phylogeography of mitochondrial L3f haplogroup

**DOI:** 10.1186/1471-2148-9-63

**Published:** 2009-03-23

**Authors:** Viktor Černý, Verónica Fernandes, Marta D Costa, Martin Hájek, Connie J Mulligan, Luísa Pereira

**Affiliations:** 1 Archaeogenetics Laboratory, Institute of Archaeology of the Academy of Sciences of the Czech Republic, Prague, The Czech Republic; 2 Instituto de Patologia e Imunologia Molecular da Universidade do Porto, (IPATIMUP), R. Dr. Roberto Frias s/n 4200-465 Porto, Portugal; 3 Department of Anthropology, University of Florida, Gainesville, FL, USA 32610-3610; 4 Medical Faculty, University of Porto, 4200-319 Porto, Portugal

## Abstract

**Background:**

Chad Basin, lying within the bidirectional corridor of African Sahel, is one of the most populated places in Sub-Saharan Africa today. The origin of its settlement appears connected with Holocene climatic ameliorations (aquatic resources) that started ~10,000 years before present (YBP). Although both Nilo-Saharan and Niger-Congo language families are encountered here, the most diversified group is the Chadic branch belonging to the Afro-Asiatic language phylum. In this article, we investigate the proposed ancient migration of Chadic pastoralists from Eastern Africa based on linguistic data and test for genetic traces of this migration in extant Chadic speaking populations.

**Results:**

We performed whole mitochondrial genome sequencing of 16 L3f haplotypes, focused on clade L3f3 that occurs almost exclusively in Chadic speaking people living in the Chad Basin. These data supported the reconstruction of a L3f phylogenetic tree and calculation of times to the most recent common ancestor for all internal clades. A date ~8,000 YBP was estimated for the L3f3 sub-haplogroup, which is in good agreement with the supposed migration of Chadic speaking pastoralists and their linguistic differentiation from other Afro-Asiatic groups of East Africa. As a whole, the Afro-Asiatic language family presents low population structure, as 92.4% of mtDNA variation is found within populations and only 3.4% of variation can be attributed to diversity among language branches. The Chadic speaking populations form a relatively homogenous cluster, exhibiting lower diversification than the other Afro-Asiatic branches (Berber, Semitic and Cushitic).

**Conclusion:**

The results of our study support an East African origin of mitochondrial L3f3 clade that is present almost exclusively within Chadic speaking people living in Chad Basin. Whole genome sequence-based dates show that the ancestral haplogroup L3f must have emerged soon after the Out-of-Africa migration (around 57,100 ± 9,400 YBP), but the "Chadic" L3f3 clade has much less internal variation, suggesting an expansion during the Holocene period about 8,000 ± 2,500 YBP. This time period in the Chad Basin is known to have been particularly favourable for the expansion of pastoralists coming from northeastern Africa, as suggested by archaeological, linguistic and climatic data.

## Background

Africa is the site of the greatest human migrations [1] both in terms of quantity and geographic range, but the extent of this influence is not reflected in current genetic surveys. To date, most studies of migrations within Africa have focused almost exclusively on the Bantu migration, by which Bantu languages together with some cultural innovations were distributed throughout Central, Eastern and Southern Africa from the present Cameroon-Nigerian border. Genetic consequences of this gene flow have been well documented in present populations [2-5]. However, the Bantu language-gene spread is likely not the only dispersal to have left genetic traces in African extant populations.

**Figure 1:**
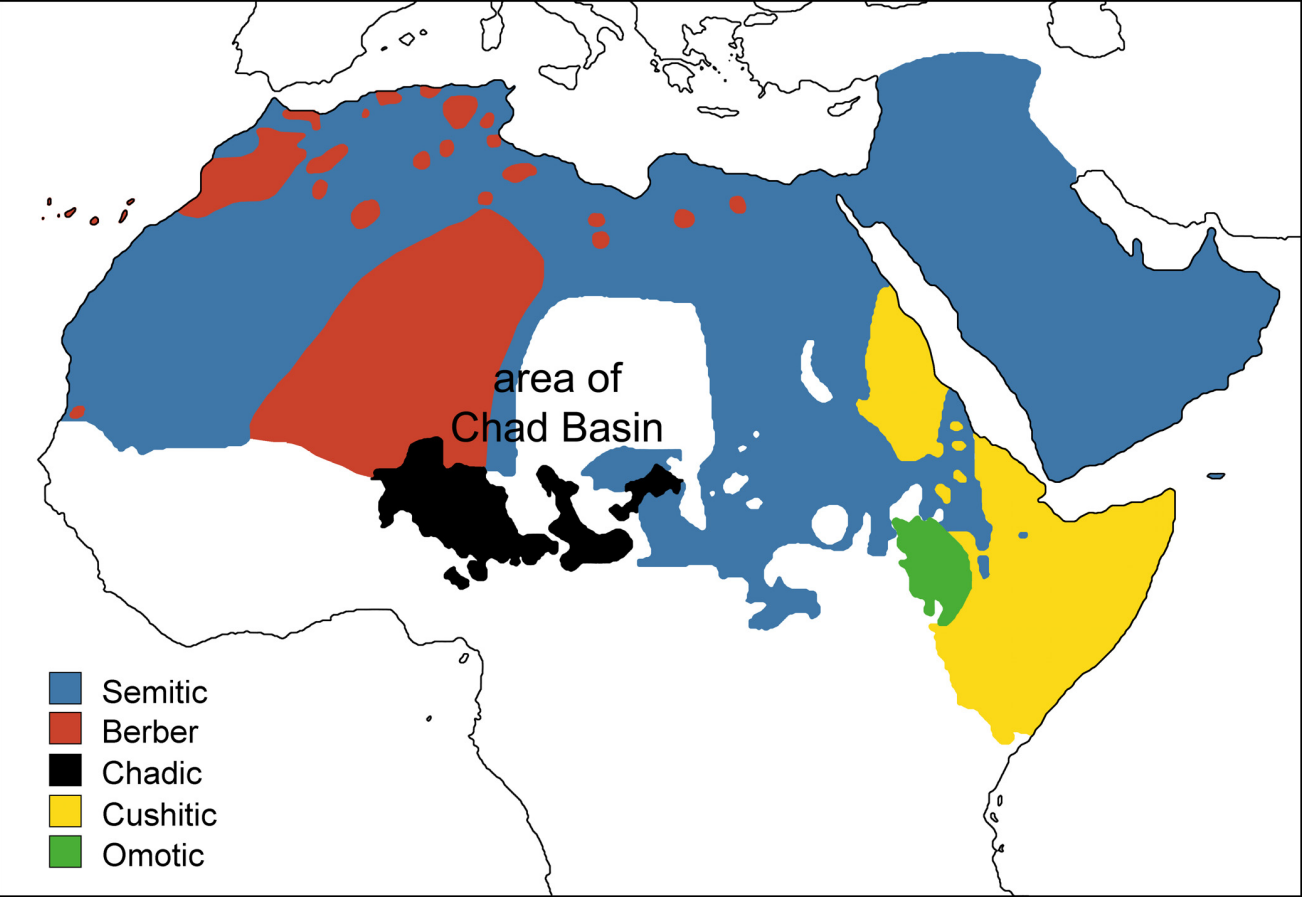
**Distribution map of the branches of the Afro-Asiatic language family**. All extant language branches are depicted; however, the Omotic branch was not implemented in the genetic study as no sequences were available to the authors.

Another important dispersal within Sub-Saharan Africa was the migration of Chadic speaking pastoralists, which has been well studied by linguists and archaeologists but not by geneticists. Chadic is one of the few well established branches of the complex Afro-Asiatic language phylum and is mainly spoken in West-Central Africa (Chad, Northern Cameroon, Northern Nigeria and Southeastern Niger) in an area centered around Lake Chad (Figure 1). It is considered the most diversified, with ~150 languages, and perhaps the most ancient subgroup within the Afro-Asiatic phylum [6,7]. Within the Afro-Asiatic family, some authors relate Chadic to Cushitic, recognising a common Cushitic-Chadic node [7], but this classification remains controversial.

According to linguistic analyses of Afro-Asiatic branches, the common ancestors of extant Chadic and Cushitic peoples inhabited East or Northeast Africa ~7,000–8,000 years before present (YBP) [8,6]. Subsequently, probably in connection with progressive desertification of the Sahara [9] and increased herding of livestock, two different migrations spread out of the "Cushitic-Chadic motherland". The westward migration (~7,000 YBP) contributed to the separation and diversification of the Chadic languages in Chad Basin, while the southward migration (~5,000 YBP) contributed to the diversification of Cushitic languages in Eastern Africa [10,8,6].

Since more than 2,000 kilometers separate the putative proto-Afro-Asiatic homeland in Northeastern Africa from Lake Chad and there are no Chadic or Cushitic speaking populations living in between these regions, some authors have proposed diverse hypotheses to explain the process by which ancient Chadic speaking peoples arrived to the Lake Chad region. Ehret [8] suggested a two step migration model; proto-Chadic people expanded westward to the northern half of the Sahara and later migrated southward through the central Sahara into the Lake Chad Basin. The "Inter-Saharan hypothesis" was developed by Blench [6], who suggested that Chadic speaking herders left their Cushitic-Chadic motherland in the Nile Valley and wandered through the large dry river system of Wadi Howar towards Lake Chad. This second hypothesis is documented linguistically by similarities of words for domesticated animals shared in Cushitic and Chadic and also by numerous archaeological findings. Some archaeological finds are located in Western Sudan, such as the so-called "*leiterband*" pottery that shows tight connections to Neolithic Khartoum [11,12] and were found at several sites along the Wadi Howar where the ancient pastoralists camped.

When Chadic speaking herders arrived at Lake Chad, they were certainly not the first inhabitants of the area. From the beginning of its settlement, the greater area of the Chad Basin acted as a center of gravity for neighbouring populations of both Niger-Congo and Nilo-Saharan origin [13]. Archaeological excavations around Lake Chad have documented several cultural changes tightly linked with oscillating climatic phases [14,15]. Uninterrupted settlement is associated with the Early Holocene (~8,000-5,000 YBP) and the establishment of the first settled communities [16-18,13]. The earliest evidence for settlement is in Dufuna in the Upper Yobe valley along the Komadugu Guna River in Northern Nigeria where the oldest boat (~8,000 YBP) on the African continent has been unearthed [19]. More information comes from Konduga, also in Nigeria and dated to 8,340 ± 250 YBP, which has yielded Neolithic artefacts such as potsherds decorated with combed incisions and polished stone industry. Additional archaeological findings, such as terracotta figurines of *Bos sp*. and bones of goats and sheep and even domesticated cattle clearly demonstrate the presence of animal husbandry [20,21]. However, there is no archaeological evidence concerning the nature of contacts between immigrating Chadic speakers and the original Sudanic farmers. Linguistic evidence, however, suggests that both groups may have cooperated in the past, i.e. Chadic loans of some basic Saharo-Sahelian terms [8].

Complete mitochondrial genome sequences are the most informative genetic data to evaluate hypotheses of past human migrations from a maternal perspective. In fact, the increased resolution of phylogenetic reconstructions and more robust estimates of the time to the most recent common ancestor (TMRCA) enabled by whole mitochondrial genome sequences have facilitated improved models of human migrations, such as Out-of-Africa [22,23], the settlement of Oceania [24-26] and the colonization of Americas [27-30].

Previous research on mitochondrial diversity in the Chad Basin (based on hypervariable segment I [HVS-I] and diagnostic coding variants) led us to identify two potential autochthonous Chad Basin mitochondrial DNA (mtDNA) haplogroups [31]. One was L3e5, with an age of 11,450 ± 3,800 years that is concordant with a Holocene expansion; this haplogroup is also seen in North African groups. The other haplogroup, tentatively named L3f2, was found almost exclusively in Chadic speaking groups with an estimated TMRCA of 28,950 ± 11,600 YBP.

The rarity of haplogroup 'L3f2' outside the Chad Basin was confirmed in a broad phylogeographical survey of 624 complete L-type genomes in Africa by [5]. Only two 'L3f2' sequences were described: one from Chad and one from Ethiopia. This work led also to some modifications in L3f nomenclature: the L3f2 in [31] was changed to L3f3 in [5] while a new subhaplogroup L3f2 was defined based on a polymorphisms at 16311, which is no longer used to define L3f, and 745iT.

A unique feature of Chadic speaking people is their unusually high frequency of L3f3 haplotypes. We identified this sub-haplogroup in 14 samples from 173 Chadic speaking individuals (8.1%) and in only 2 samples from 275 non-Chadic speaking individuals (0.7%). In this study, we perform whole genome sequence analysis of L3f3 haplotypes in Chad Basin populations in order to conduct a high-resolution characterization of the sub-haplogroup and more robust TMRCA estimation of its expansion. Specifically, we sequenced 16 different L3f haplotypes from Chad Basin, of which 14 belong to the L3f3 sub-haplogroup.

## Results

### L3f phylogeny

**Figure 2:**
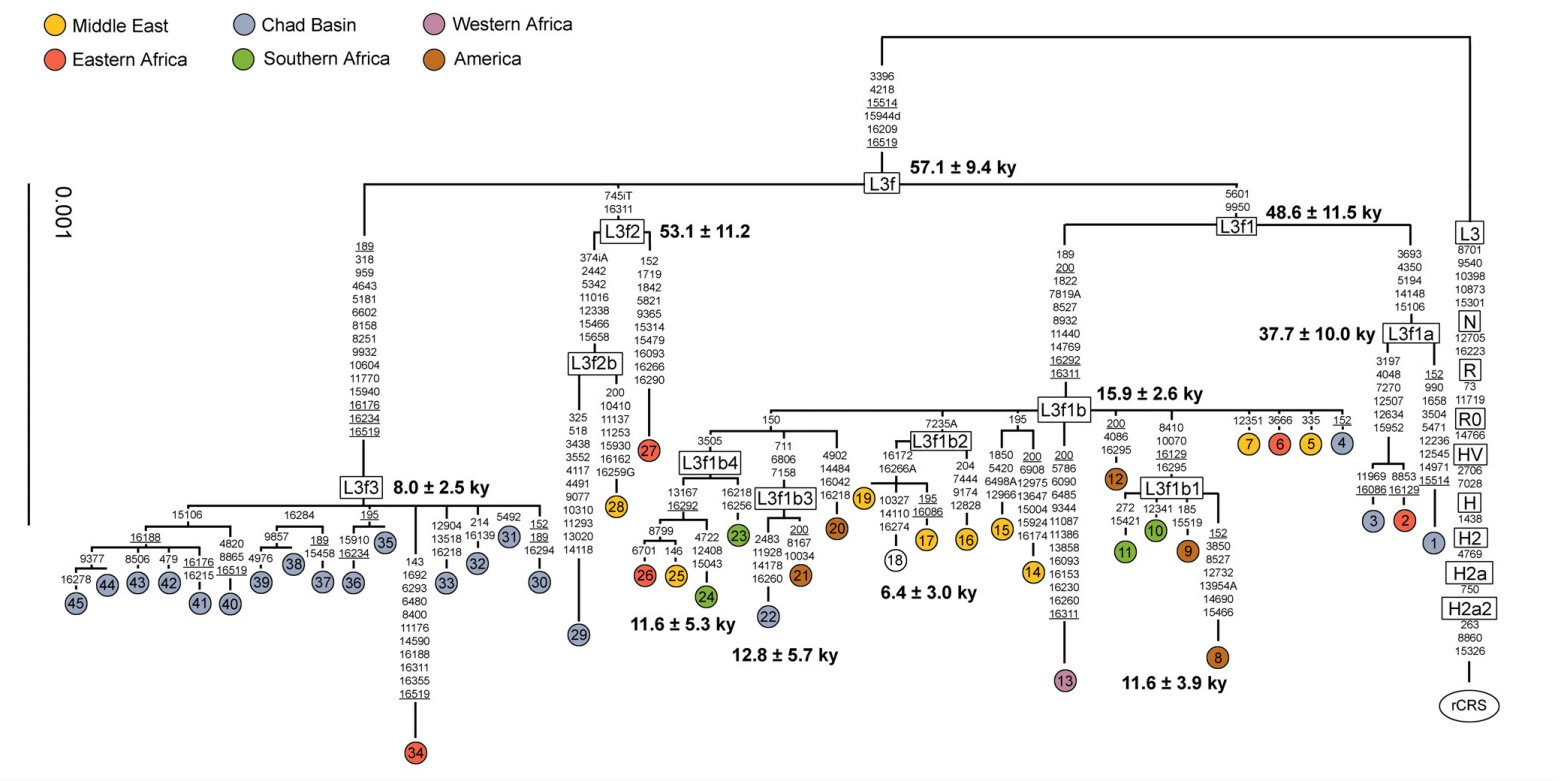
**Tree of 45 mtDNA sequences belonging to haplogroup L3f**. The tree is rooted using the revised Cambridge reference sequence (rCRS) as an outgroup. Mutations are shown on the branches; they are transitions unless a base is explicitly indicated; recurrent mutations are underlined. The geographic origin is shown on the top of the figure; Accession Number and references for published sequences are in the Additional file 1.

Sixteen new mtDNA genomes from Chad Basin populations and 29 whole L3f genomes published elsewhere (see Additional file 1) were used to construct a phylogenetic tree (Figure 2) and to estimate the time to the most common ancestor (TMRCA) (Table 1). The tree confirms the main points of Behar study [5], but has important additional phylogenetic features based on the Chad Basin sequences.

**Table 1:** Control and coding region age estimates for sub-haplogroups of L3f

	Control region		Coding region	
haplogroup	TMRCA	SD	TMRCA	SD

L3f	54,600	12,100	57,100	9,400
L3f1	---	---	48,600	11,500
L3f1a	---	---	37,700	10,000
L3f1b	28,900	4,700	15,900	2,600
L3f1b1	9,400	4,000	11,600	3,900
L3f1b2	---	---	6,400	3,000
L3f1b3	---	---	12,800	5,700
L3f1b4	---	---	11,600	5,300
L3f2	---	---	53,100	11,200
L3f3	15,900	7,500	8,000	2,500

Haplogroup L3f is defined by the coding variants 3396-4218-15514-15944del and the control region motif 16209–16519 with a TMRCA of 57,100 ± 9,400 YBP. This haplogroup diversifies into sub-haplogroups L3f1, L3f2 and L3f3. The most geographically widespread sub-haplogroup is L3f1, which is distributed across the African continent [3] and also Arabia [32,33] and has a TMRCA of 48,600 ± 11,500 YBP. In our tree, sub-haplogroup L3f1 as defined by Salas et al. [3] using HVS-I data is now represented by two sub-haplogroups, ancestral L3f1 and derived L3f1b that carries two control region variants 16292–16311 [3] as well as six coding variants. The long branch leading to L3f1b may indicate constant population size and/or strong genetic drift throughout the dry climatic conditions during the last glacial period. On the other hand, the recent age for sub-haplogroup L3f1b of 15,900 ± 2,600 YBP (or 15,000 ± 3,000 YBP if not including the divergent sequence number 13) and its star-like phylogeny suggests a population expansion in the African population bearing its ancestral motif during the climatic improvement after the Last Glacial Maximum (LGM). Subsequently, other L3f1b sub-haplogroups emerged in the Holocene; L3f1b1 11,600 ± 3,900 YBP, L3f1b2 6,400 ± 3,000 YBP, L3f1b3 12,800 ± 5,700 YBP and L3f1b4 11,600 ± 5,300 YBP. The youngest clade, L3f1b2, seems to be more frequent in the Middle East. L3f1a seems to be older (37,700 ± 10,000 YBP) than its sister sub-haplogroup L3f1b and is also less diversified. A few samples from Chad belong to these sub-haplogroups: two to L3f1a and one to L3f1b3.

Sub-haplogroup L3f2 is only defined by the highly recurrent control region variant 16311 and insertion 745T. However, it contains very divergent lineages resulting in a very old age for this sub-haplogroup (53,100 ± 11,200 YBP). One L3f2b sample was observed in Chad.

The clearest difference between the tree presented here and the L3f portion of the Sub-Saharan L-tree depicted in [5] is the "Chad Basin" clade L3f3. Robusticity of this clade is unambiguously supported by 10 coding variants. The TMRCA of this sub-haplogroup is 8,000 ± 2,500 YBP, an estimate concordant with both archaeological and linguistic data. The only non-Chad Basin sequence in this subhaplogroup is from Ethiopia, although it has a very divergent sequence suggesting it is evolutionarily distinct from the Chad Basin sequences; the TMRCA estimate for L3f3 without this Ethiopian sequence is 6,500 ± 2,500 YBP.

In order to obtain additional information on L3f frequency distributions, we examined the current dataset of 5,046 published African HVS-I mtDNA sequences (Additional file 2) and identified 315 individuals with L3f sequences for a total of 129 different haplotypes. We constructed a network based on these sequences (Additional file 3) and were only able to identify sub-haplogroups L3f1b, L3f1b1 and L3f3 (with some recurrence) because of the lower resolution of HVS-I compared to whole genome sequences. Nevertheless, the higher frequency of L3f3 in Chadic speaking individuals is confirmed and the TMRCA of the L3f ancestral motif (54.6 ± 12.1 kya) is in good agreement with that obtained by Behar et al. [5] (see their Supporting figure 1) and with our dates (see Figure 2 and Table 1). Again, as in [31], HVS-I age estimate for L3f3 is older (15.9 ± 7.5 kya excluding recurrent and 22.3 ± 7.1 kya including recurrent sites) than the one obtained based on the coding-region information. However, the network based on the expanded Africa dataset suggests a possible new haplotype defined by the motif 16278-16294-16301-16354. Tentatively, this cluster can be dated to 10,900 ± 4,100 years ago, so it chronologically corresponds with most of the Holocene L3f1b clades and with the Chadic L3f3 as well. This sub-haplogroup seems to be frequent in Chadic and non-Chadic speaking populations of Central Africa and northeastern Africa.

### Population structure

Analyses of molecular variance (AMOVA) and multidimensional scaling (MDS) analyses can provide further insight into the genetic diversification of Chad Basin populations, allowing us to compare geography and language as the main influences on population structure. First, we tested the influence of linguistic affiliation on HVS-I mtDNA variability by grouping Chad Basin and other Afro-Asiatic populations living outside this area (a total of 44 populations) according to language groups (Semitic, Berber, Chadic, Cushitic, Nilo-Saharan, and Niger-Congo). We observed that only 3.4% of variation was found among linguistic groups and only 4.0% within groups. Similarly, when these populations were grouped according to their geographic location (North Africa, Chad Basin, Ethiopia, and Tanzania), the distribution of variability proportions was 5.5% among regional groups and 3.0% within groups. These results show a similarly low effect of language and geography in determining population structure in and around the Chad Basin. With both linguistic and geographic groupings, the vast majority of mitochondrial variation (about 92%) partitioned within populations, testifying to a low level of population structure.

**Figure 3:**
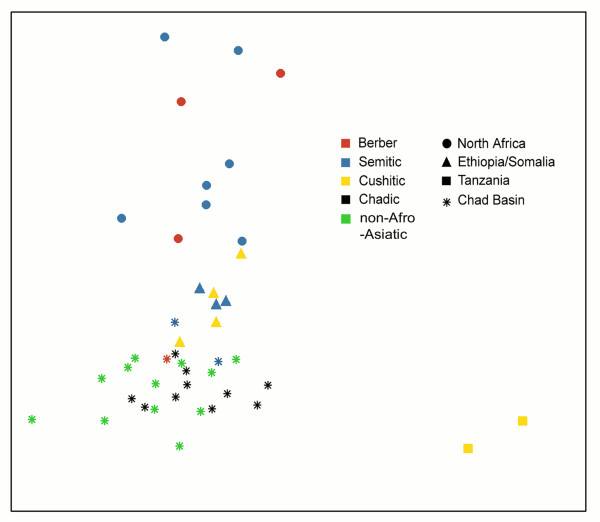
**Multidimensional scaling of F_ST _distances based on HVS-I sequences of Chad Basin populations and their Afro-Asiatic neighbours**.

We then estimated pairwise F_ST _genetic distances between populations (Additional file 4) and displayed these on a MDS plot (Figure 3). Interesting results are immediately evident – while Chadic populations form a relatively homogeneous group, the Cushitic populations split into two completely different clusters. The first group is composed of Horn of African populations, such as Ethiopian and Somali Cushitic populations, which are close to neighbouring Ethiopian Semitic speaking groups and relatively close also to Chadic people from the Chad Basin. The second Cushitic group is composed by more southern groups from Tanzania, i.e. Burunge and Iraqw, who occupy outlier positions even within the Afro-Asiatic MDS plot. In the MDS plot, geography is more strongly associated with genetic distance than is linguistic affiliation. Overall, we observe that Chadic speaking populations are intermixed with other populations from Chad Basin, including Niger-Congo, Semitic, and Berber speaking people. In this context, it seems that the linguistic categories play a secondary role in structuring the genetic diversity.

## Discussion

We use high-resolution genetic data to investigate the genetic and linguistic support for hypotheses concerning the population history in the Chad Basin. The mitochondrial L3f3 haplogroup is found almost exclusively in Chadic speaking populations and its TMRCA corresponds well with archaeological and linguistic dates of the proposed migration of Chadic speaking pastoralists from East or North East Africa to the Chad Basin.

Our TMRCA estimates (Table 1) and signatures of population expansions (Figure 2) for L3f1b and L3f3 appear to correlate well with the paleoclimatic record in African Sahel, another highly overlooked data source in African genetic studies. This omission is surprising as the Sahara is a region of dramatic environmental changes, with many oscillatory phases between arid-uninhabitable desert and humid-fertile landscape of lakes and savannah. These changes are driven by glacial cycles, with glacial conditions in the northern hemisphere being associated with cold and arid conditions over northern Africa (reviewed in [34]). Our results suggest that following the emergence of L3f in East Africa soon after the Out-of-Africa migration [5] around 57,100 ± 9,400 YBP, genetic drift and/or demographic decline occurred throughout the dryer, second part of Late Pleistocene. This conclusion can be inferred from the very long branches leading to many of the L3f sub-haplogroups. A dryer period in the latter part of the Late Pleistocene is supported by fossil evidence showing that during the LGM, some 21,000 YBP, the Sahara desert covered a much larger area than today [34].

Subsequently, in the post-Last Glacial Maximum when climatic conditions had improved, an opportunity for population expansion and consequent mutation fixation again occurred, detectable mainly in our L3f1b data. L3f1b is widespread throughout Africa and has also dispersed into the Middle East with evidence of several new Holocene-emerging sub-haplogroups. In fact, the Sahara was repopulated during the first half of the Holocene when humid conditions and greening were established by ~10,000 YBP, leading to the Holocene Climatic Optimum. The Optimum lasted till ~6,000 YBP, when the shift towards more permanent aridity occurred culminating with the formation of the current Sahara Desert. Additionally, several lines of evidence suggest that a linguistically distinct part of the East African population migrated to the climatically suitable Chad Basin; specifically, our data demonstrate that the Chadic-specific L3f3 clade expanded during the Holocene. The age for L3f3 clade, based on mtDNA coding region diversity (8.0 ± 2.5 kya), is remarkably concordant with archaeological findings for the proto-Chadic migration. This clade might be then considered as a strong genetic signature of the Chadic migration, which is highly correlated with paleoclimatic conditions. We can parallel the role played by L3f sub-haplogroups in climatic-related human population expansions in the Sahara/Sahel region with the western Eurasian sub-haplogroups V [35], H1 and H3 [36-38]. These haplogroups are European genetic signs of a re-settlement of the continent from an Iberian refugium, after the improvement of climatic conditions post-Last Glacial Maximum. As phylogenetic inferences improve, genetic signs of population contractions and expansions related with broad intense climatic changes are expected to be detected.

## Conclusion

We provide genetic support for an Early Holocene migration within Africa. A high-resolution phylogeny of haplogroup L3f based on whole mitochondrial genome sequences shows several clades that are unevenly distributed throughout Africa and Near East. Specifically, clade L3f3 is geographically limited to the Chad Basin where it reaches high frequencies especially in Chadic-speaking groups while almost absent in Niger-Congo and Nilo-Saharan people. Within the Afro-Asiatic language phylum, the Chadic branch is linguistically close to the East African Cushitic branch although they are separated by ~2,000 km of territory in which different Semitic and Nilo-Saharan peoples live today. We show that only northern Cushitic groups from Ethiopia and Somalia are genetically close to Chadic populations. Thus, the archaeologically and linguistically supported route of proto-Chadic pastoralists via Wadi Howar to the Chad Basin may have genetic support. Moreover, our molecular genetic date for the Chadic-specific L3f3 clade is consistent with the suggested Holocene dispersal.

## Methods

### Whole genome sequencing of L3f3

In two previous publications [39,31], HVS-I sequence analysis of five Chadic-speaking populations from the Chad Basin was performed. Study populations included those from northern Cameroon such as the Hide and the Mafa living in the Mandara Mountains (*N *= 23 and *N *= 32, respectively), the Kotoko of the Shari basin (*N *= 56), the Masa of the Logon basin (*N *= 32) and the Buduma of the northwestern shore and islands of Lake Chad in Niger (*N *= 30). All populations speak their own language broadly classified as Central Chadic branch within Afro-Asiatic language phylum. Sequences were classified into haplogroups, following specifications described in [31], leading to the identification of a high proportion of L3f haplotypes (defined by the sequence mutation motif 16209–16223 when compared to the revised Cambridge Reference Sequence or rCRS [40].

In the present study, a total of 16 different L3f haplotypes from the Chad Basin L3f sequences were chosen for whole genome sequencing (accession numbers FJ625845–FJ625860) using 32 primers (see [41] for primer sequences and PCR specifications). Amplicons were purified and sequenced using the forward primers. In some cases (e.g. the poly-C stretches between nts 568–573 and 16184–16193), the reverse primer was used. Sequencing was performed on a ABI 3100 DNA Analyzer (Applied Biosystems, Forster City, CA). Chromatograms were evaluated by two independent observers (MC and LP) with the help of SeqScape (Applied Biosystems) and BioEdit version 7.0.4.1 [42]. In cases of ambiguous results, new PCR amplification and sequencing reactions were performed.

### Phylogenetic analysis

A L3f phylogenetic tree was constructed based on the new 16 Chad sequences and 29 previously published whole L3f genomes (see Additional file 1). A preliminary network analysis [43] and information published in [5] led to a suggested branching order for the tree. Projected shape of phylogenetic tree (estimation of the length of the branches) was tested by means of PAML 3.13 [44], assuming the HKY85 mutation model with gamma-distributed rates (approximated by a discrete distribution with 32 categories). For calculation of the time to the most recent ancestor (TMRCA) for specific clades in the phylogeny, ρ statistics (mean divergence from inferred ancestral haplotype) were used with a coding region (nts 577–16023) mutation rate estimate of one transition per 5138 years [45]. Standard errors were calculated as in [46].

### HVS-I networks

We also constructed a L3f network using HVS-I sequences (nts 16090–16365) that carried variant 16209 chosen from an expanded African population database listed in the Additional file 2 (all L0a2 sequences with 16209 were discarded). Networks were constructed using the reduced median algorithm (Network 4.5.0.0), followed by the median joining algorithm to resolve intermediate nodes [43]. For the total dataset of 315 haplotypes, a reducing factor of one was used, instead of the default value of two [43]. For calculation of the TMRCA, ρ statistics were used with a HVS-I mutation rate of one transition per 20,180 years [47]. The standard deviation of the ρ estimator was calculated according to [46].

### Population structure

Analysis of population structure was calculated using Arlequin software version 3.0 [48]. For AMOVA, computations were based on haplotype frequencies. Two groupings were performed: (1) by language branches and (2) by geographic regions. F_ST _genetic distances for all populations in the Additional file 2 as well as for a subset of Afro-Asiatic speaking populations and populations inhabiting Chad Basin were calculated using HVS-I mtDNA sequences. F_ST _distances were transformed to Slatkin linearized form and visualized by non-metric multidimensional scaling (MDS) using PROXSCAL included in the SPSS 10.0 software (SPSS Inc, Chicago, IL, USA).

## Authors' contributions

VČ has made substantial contributions to conception and design, acquisition of data, interpretation of the results and drafting the manuscript. LP has been involved in the analysis of whole genome sequencing, phylogenetic analyses and interpretation of the data and critically revised the manuscript. VF and MDC performed the whole genome sequencing and participated in the phylogenetic analyses. MH provided population genetic analyses and its interpretation. CJM assisted with interpretation of the data and revision of the manuscript. All authors read and approved the final version of the manuscript.

## Supplementary Material

Additional file 1 **Samples used for the whole genome L3f phylogeny**. List of DNA haplotypes used to construct the L3f phylogeny (16 new sequences and 29 previously published sequences). Click here for file

Additional file 2 **Population samples used for the study of mtDNA differentiation**. List of populations used in the current study. Click here for file

Additional file 3 **Reduced median network relating L3f sequences**. Reduced median network of L3f sequences. The central motif (star) differs from rCRS at position 16209 in HVS-I control region. Numbers along links refer to nucleotide positions minus 16000. Size of the nodes is proportional to the number of sequences included. Only selected mutations are shown. Click here for file

Additional file 4 **Matrix of F_ST _between populations**. Matrix of F_ST _values derived from mtDNA HVS I sequences in African populations speaking Afro-Asiatic languages and the non-Afro-Asiatic speaking populations of Chad Basin; F_ST _values below diagonal and significant values (p < 0.001) above diagonal. Click here for file
