# Supplementary material for: Migration of Chadic speaking pastoralists within Africa based on population structure of Chad Basin and phylogeography of mitochondrial L3f haplogroup
Source: BMC Evol Biol. 2009 Mar 23;9:63. doi: 10.1186/1471-2148-9-63 (PMC2680838; doi:10.1186/1471-2148-9-63)
Supplement: Additional file 1 — Samples used for the whole genome L3f phylogeny. List of DNA haplotypes used to construct the L3f phylogeny (16 new sequences and 29 previously published sequences). [file 1471-2148-9-63-S1.doc]

**Additional file 1**

Samples used for the whole genome L3f phylogeny

| Designation | Accession Number/Original Serial | Geographic origin | Haplogroup | Reference |
| --- | --- | --- | --- | --- |
| L1 | EU092891 | Chad | L3f1a | 2 |
| L2 | DQ341078 | Ethiopia | L3f1a | 3 |
| L3 | FJ625845 | Chad | L3f1a | 1 |
| L4 | FJ625860 | Chad | L3f1b | 1 |
| L5 | EU092771 | Egypt | L3f1b | 2 |
| L6 | DQ341077 | Ethiopia | L3f1b | 3 |
| L7 | EU092741 | Syria | L3f1b | 2 |
| L8 | 578 | USA | L3f1b1 | 4 |
| L9 | 580 | USA | L3f1b1 | 4 |
| L10 | EU092696 | Mozambique | L3f1b1 | 2 |
| L11 | EU092857 | South Africa | L3f1b1 | 2 |
| L12 | 579 | USA | L3f1b | 4 |
| L13 | EU092732 | Guinea-Bissau | L3f1b | 2 |
| L14 | EU092680 | Israel | L3f1b | 2 |
| L15 | EU092751 | Lebanon | L3f1b | 2 |
| L16 | EU092805 | Yemen | L3f1b2 | 2 |
| L17 | EU092758 | Jordan | L3f1b2 | 2 |
| L18 | EU092953 | Unknown | L3f1b2 | 2 |
| L19 | EU935451 | Egypt | L3f1b2 | 5 |
| L20 | 584 | USA | L3f1b | 4 |
| L21 | 581 | USA | L3f1b3 | 4 |
| L22 | EU092883 | Chad | L3f1b3 | 2 |
| L23 | EU092704 | Mozambique | L3f1b4 | 2 |
| L24 | EU092865 | South Africa | L3f1b4 | 2 |
| L25 | EU092791 | Oman | L3f1b4 | 2 |
| L26 | EU092912 | Kenya | L3f1b4 | 2 |
| L27 | DQ341076 | Ethiopia | L3f2a | 3 |
| L28 | EU092770 | Egypt | L3f2b | 2 |
| L29 | EU092877 | Chad | L3f2b | 2 |
| L30 | FJ625856 | Chad | L3f3 | 1 |
| L31 | FJ625848 | Chad | L3f3 | 1 |
| L32 | FJ625849 | Chad | L3f3 | 1 |
| L33 | FJ625853 | Chad | L3f3 | 1 |
| L34 | DQ341075 | Ethiopia | L3f3 | 3 |
| L35 | FJ625847 | Chad | L3f3 | 1 |
| L36 | FJ625846 | Chad | L3f3 | 1 |
| L37 | FJ625854 | Chad | L3f3 | 1 |
| L38 | FJ625855 | Chad | L3f3 | 1 |
| L39 | EU092894 | Chad | L3f3 | 2 |
| L40 | FJ625851 | Chad | L3f3 | 1 |
| L41 | FJ625857 | Chad | L3f3 | 1 |
| L42 | FJ625852 | Chad | L3f3 | 1 |
| L43 | FJ625859 | Chad | L3f3 | 1 |
| L44 | FJ625850 | Chad | L3f3 | 1 |
| L45 | FJ625858 | Chad | L3f3 | 1 |

1) This work; 2) Behar et al. 2008; 3) Torroni et al. 2006; 4) Howell et al. 2004; 5) Kujanova et al. (in press)
