# Supplementary material for: Migration of Chadic speaking pastoralists within Africa based on population structure of Chad Basin and phylogeography of mitochondrial L3f haplogroup
Source: BMC Evol Biol. 2009 Mar 23;9:63. doi: 10.1186/1471-2148-9-63 (PMC2680838; doi:10.1186/1471-2148-9-63)
Supplement: Additional file 2 — Population samples used for the study of mtDNA differentiation. List of populations used in the current study. [file 1471-2148-9-63-S2.doc]

**Additional file 2**

Population samples used for the study of mtDNA differentiation

| **Population** | **Code** | ***n*** | **Location** | **Language Family** | **Language Branch** | **Reference** |
| --- | --- | --- | --- | --- | --- | --- |
| Egyptians | EHA | 35 | Egypt | Afro-Asiatic | Semitic | 1 |
| Burunge | BUR | 38 | Tanzania | Afro-Asiatic | Cushitic | 2 |
| Datoga | DA2 | 39 | Tanzania | Nilo-Saharan | Eastern Sudanic | 2 |
| Hadza | HA2 | 79 | Tanzania | Khoisan | Hatsa | 2 |
| Sukuma | SU2 | 11 | Tanzania | Niger-Congo | Atlantic-Congo | 2 |
| Sandawe | SAN | 82 | Tanzania | Khoisan | Sandawe | 2 |
| Turu | TRU | 29 | Tanzania | Niger-Congo | Atlantic-Congo | 2 |
| Hadza | HA1 | 49 | Tanzania | Khoisan | Hatsa | 3 |
| Datoga | DA1 | 18 | Tanzania | Nilo-Saharan | Eastern Sudanic | 3 |
| Iraqw | IRQ | 12 | Tanzania | Afro-Asiatic | Cushitic | 3 |
| Sukuma | SU1 | 21 | Tanzania | Niger-Congo | Atlantic-Congo | 3 |
| Turkana | TUK | 37 | Kenya | Nilo-Saharan | Eastern Sudanic | 4 |
| Somali | SOM | 27 | Kenya | Afro-Asiatic | Cushitic | 4 |
| Kikuyu | KIK | 25 | Kenya | Niger-Congo | Atlantic-Congo | 4 |
| Bantu | BAN | 307 | Mozambique | Niger-Congo | Atlantic-Congo | 5 |
| Nuba | NBA | 14 | Sudan | mixed | ?? | 6 |
| Nilotic | NIL | 19 | Sudan | Nilo-Saharan | ??Eastern Sudanic | 6 |
| Dinka | DIN | 46 | Sudan | Nilo-Saharan | Eastern Sudanic | 6 |
| Hide | HID | 23 | Cameroon | Afro-Asiatic | Chadic | 7 |
| Kotoko | KOT | 56 | Cameroon | Afro-Asiatic | Chadic | 7 |
| Mafa | MAF | 32 | Cameroon | Afro-Asiatic | Chadic | 7 |
| Masa | MAS | 32 | Cameroon | Afro-Asiatic | Chadic | 7 |
| Buduma | BUD | 30 | Niger | Afro-Asiatic | Chadic | 7 |
| Arabs Chad | ACH | 27 | Chad | Afro-Asiatic | ?? | 7 |
| Arabs Shuwa | ASH | 38 | Nigeria | Afro-Asiatic | ?? | 7 |
| Fali | FA2 | 40 | Cameroon | Niger-Congo | Atlantic-Congo | 7 |
| Fulani | FBO | 49 | Chad | Niger-Congo | Atlantic-Congo | 7 |
| Fulani | FTC | 40 | Cameroon | Niger-Congo | Atlantic-Congo | 7 |
| Fulani | FTI | 47 | Burkina Faso | Niger-Congo | Atlantic-Congo | 8 |
| Fulani | FBA | 50 | Burkina Faso | Niger-Congo | Atlantic-Congo | 8 |
| Kanembou | KAN | 50 | Chad | Nilo-Saharan | Saharan | 7 |
| Kanuri | KA2 | 31 | Nigeria | Nilo-Saharan | Saharan | 7 |
| Hausa | HAU | 20 | Niger/Nigeria | Afro-Asiatic | Chadic | 7 |
| Kanuri | KA1 | 14 | Niger/Nigeria | Nilo-Saharan | Saharan | 7 |
| Fulani | FUL | 61 | Niger/Nigeria | Niger-Congo | Atlantic-Congo | 7 |
| Songhai | SON | 10 | Niger | Nilo-Saharan | Songhai | 7 |
| Tuareg | TUA | 26 | Niger | Afro-Asiatic | Berber | 7 |
| Yoruba | YOR | 33 | Nigeria | Niger-Congo | Atlantic-Congo | 7 |
| Mandenka | MAN | 79 | Senegal | Niger-Congo | Mande | 9 |
| Egypt Lower | EGL | 59 | Egypt | Afro-Asiatic | ? Semitic | 6 |
| Egypt Upper | EG1 | 35 | Egypt | Afro-Asiatic | ? Semitic | 6 |
| Egypt Upper | EG2 | 58 | Egypt | Afro-Asiatic | ? Semitic | 10 |
| Nubians | NUB | 78 | Sudan/Egypt | Nilo-Saharan | Eastern Sudanic | 6 |
| Arabs | MOR | 32 | Morocco | Afro-Asiatic | ? Semitic | 11 |
| Berbers | BEM | 60 | Morocco | Afro-Asiatic | ? Berber | 11 |
| Mauritanians | MAU | 30 | Mauritania | Afro-Asiatic | ? Semitic | 11 |
| Sereer | SER | 23 | Senegal | Niger-Congo | Atlantic-Congo | 7 |
| Wolof | WOL | 48 | Senegal | Niger-Congo | Atlantic-Congo | 7 |
| Saharawi | SAH | 25 | West Sahara | Afro-Asiatic | ? Semitic | 11 |
| Berbers Souss | BES | 50 | Morocco | Afro-Asiatic | Berber | 12 |
| Kesra | KES | 47 | Tunisia | Afro-Asiatic | ? | 13 |
| Zriba | ZRI | 50 | Tunisia | Afro-Asiatic | ? | 13 |
| Amhara | AM1 | 74 | Ethiopia | Afro-Asiatic | Semitic | 14 |
| Amhara | AM2 | 120 | Ethiopia | Afro-Asiatic | Semitic | 15 |
| Tigrais | TIG | 53 | Ethiopia/Eritrea | Afro-Asiatic | Cushitic | 15 |
| Oromo | ORO | 33 | Ethiopia | Afro-Asiatic | Cushitic | 15 |
| Gurage | GUR | 21 | Ethiopia | Afro-Asiatic | Semitic | 15 |
| Afars | AFA | 16 | Ethiopia | Afro-Asiatic | Cushitic | 15 |
| Guinea | GUA | 50 | Guinea-Bissau | Niger-Congo | ? | 16 |
| Guinea | GUB | 22 | Guinea-Bissau | Niger-Congo | ? | 16 |
| Guinea | GUC | 62 | Guinea-Bissau | Niger-Congo | ? | 16 |
| Guinea | GUD | 77 | Guinea-Bissau | Niger-Congo | ? | 16 |
| Guinea | GUE | 77 | Guinea-Bissau | Niger-Congo | ? | 16 |
| Guinea | GUF | 58 | Guinea-Bissau | Niger-Congo | ? | 16 |
| Guinea | GUG | 26 | Guinea-Bissau | Niger-Congo | ? | 16 |
| Bakaka | CBA | 50 | Cameroon, south | Niger-Congo | Atlantic-Congo | 17 |
| Bamileke | CBM | 48 | Cameroon, south | Niger-Congo | Atlantic-Congo | 17 |
| Bassa | CBS | 46 | Cameroon, south | Niger-Congo | Atlantic-Congo | 17 |
| Daba | DAB | 20 | Cameroon, north | Afro-Asiatic | Chadic | 17 |
| Ewondo | EWO | 53 | Cameroon, south | Niger-Congo | Atlantic-Congo | 17 |
| Fali | FA1 | 41 | Cameroon, north | Niger-Congo | Atlantic-Congo | 17 |
| Fulani | FU2 | 34 | Cameroon, north | Niger-Congo | Atlantic-Congo | 17 |
| Mandara | MDA | 37 | Cameroon, north | Afro-Asiatic | Chadic | 17 |
| Ouldeme | OUL | 28 | Cameroon, north | Afro-Asiatic | Chadic | 17 |
| Podokwo | POD | 39 | Cameroon, north | Afro-Asiatic | Chadic | 17 |
| Tali | TAL | 22 | Cameroon, north | Afro-Asiatic?? | ? | 17 |
| Tupuri | TUP | 25 | Cameroon, north | Niger-Congo | Atlantic-Congo | 17 |
| Akele | KEL | 48 | Gabon, west | Niger-Congo | Atlantic-Congo | 18 |
| Ateke | TEK | 54 | Gabon, southeast | Niger-Congo | ? | 18 |
| Babongo | BAB | 45 | Gabon, southeast | Niger-Congo | Atlantic-Congo | 18 |
| Baka | BCC | 30 | Cameroon, centre | Niger-Congo | Atlantic-Congo | 18 |
| Baka | BCW | 58 | Cameroon, southwest | Niger-Congo | Atlantic-Congo | 18 |
| Baka | BGB | 39 | Gabon, northeast | Niger-Congo | Atlantic-Congo | 18 |
| Bakola | BAK | 88 | Cameroon, west | Niger-Congo | Atlantic-Congo | 18 |
| Bakoya | BKY | 31 | Gabon, northeast | Niger-Congo | ? | 18 |
| Benga | BEN | 50 | Gabon, northwest | Niger-Congo | Atlantic-Congo | 18 |
| Biaka | BIA | 56 | CAR | Niger-Congo | ? | 18 |
| Duma | DUM | 47 | Gabon, east | Niger-Congo | Atlantic-Congo | 18 |
| Eshira | GIS | 40 | Gabon, west | Niger-Congo | Atlantic-Congo | 18 |
| Eviya | EVI | 38 | Gabon, center | Niger-Congo | ? | 18 |
| Ewondo | EWD | 25 | Cameroon, west | Niger-Congo | Atlantic-Congo | 18 |
| Fang | FCM | 39 | Cameroon, south | Niger-Congo | Atlantic-Congo | 18 |
| Fang | FGB | 66 | Gabon, north | Niger-Congo | Atlantic-Congo | 18 |
| Galoa | GAL | 51 | Gabon, west | Niger-Congo | Atlantic-Congo | 18 |
| Kota | KTA | 56 | Gabon, east | Niger-Congo | Atlantic-Congo | 18 |
| Makina | MAK | 45 | Gabon, center | Niger-Congo | ? | 18 |
| Mbuti | MBU | 39 | DRC | Nilo-Saharan | Central Sudanic | 18 |
| Mitsogo | TSO | 64 | Gabon, center | Niger-Congo | Atlantic-Congo | 18 |
| Ndumu | NDU | 39 | Gabon, southeast | Niger-Congo | Atlantic-Congo | 18 |
| Ngumba | NGU | 88 | Cameroon, west | Niger-Congo | ? | 18 |
| Nzebi | NZE | 63 | Gabon, southeast | Niger-Congo | Atlantic-Congo | 18 |
| Obamba | OBA | 47 | Gabon, southeast | Niger-Congo | Atlantic-Congo | 18 |
| Orungu | ORU | 20 | Gabon, west | Niger-Congo | Atlantic-Congo | 18 |
| Punu | PUN | 52 | Gabon, southwest | Niger-Congo | Atlantic-Congo | 18 |
| Shake | SHA | 51 | Gabon, east | Niger-Congo | Atlantic-Congo | 18 |
| Tikar | BEZ | 35 | Cameroon, north | Niger-Congo | Atlantic-Congo | 18 |
| Angolans | ANG | 44 | Angola, southwest | Niger-Congo | ? | 19 |
| Kenyans | KEN | 100 | Kenya | mixed | x | 20 |
| Sao Tomeans | STO | 50 | Sao Tome e Principe | Creole | x | 21 |
| Bubi | BUB | 45 | Equatorial Guinea | Niger-Congo | Atlantic-Congo | 21 |
|  |  | 5046 |  |  |  |  |

1) Kujanová et al. (in press); 2) Gonder et al. 2007; 3) Knight et al. 2003; 4) Watson et al. 1997; 5) Salas et al. 2002; 6) Krings et al. 1999; 7) Černý et al. 2007; 8) Černý et al. 2006; 9) Graven et al. 1995; 10) Stevanovitch et al. 2004; 11) Rando et al. 1998; 12) Brakez et al. 2001; 13) Cherni et al. 2005; 14) Thomas et al. 2002; 15) Watson et al. 1997; 16) Rosa et al. 2004; 17) Coia et al. 2005; 18) Quintana-Murci et al. 2008; 19) Plaza et al. 2004; 20) Brandstätter et al. 2004; 21) Mateu et al. 1997.
