# Supplementary figures and images for: Migration of Chadic speaking pastoralists within Africa based on population structure of Chad Basin and phylogeography of mitochondrial L3f haplogroup
Source: BMC Evol Biol. 2009 Mar 23;9:63. doi: 10.1186/1471-2148-9-63 (PMC2680838; doi:10.1186/1471-2148-9-63)

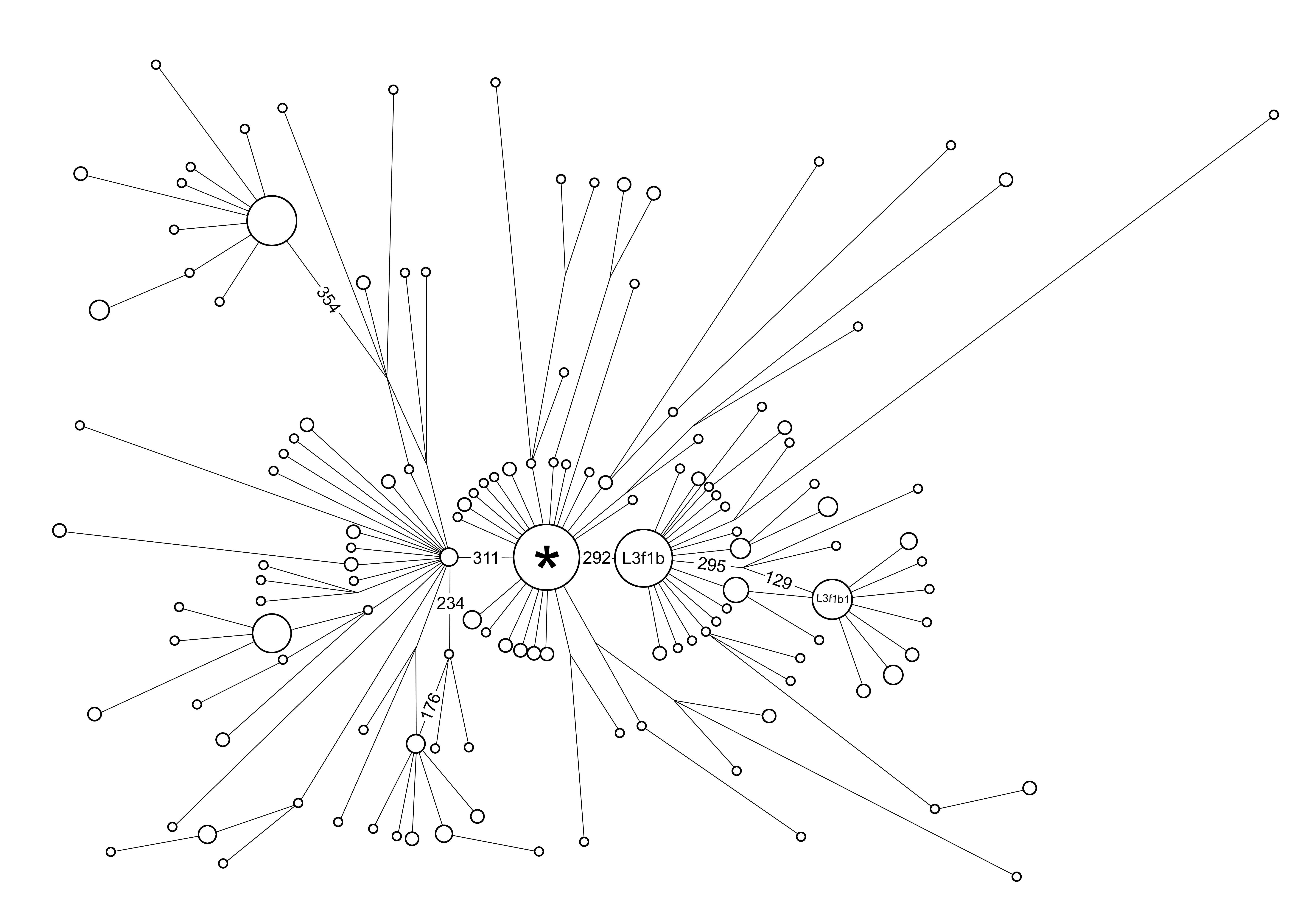

Supplement: Additional file 3 — Reduced median network relating L3f sequences. Reduced median network of L3f sequences. The central motif (star) differs from rCRS at position 16209 in HVS-I control region. Numbers along links refer to nucleotide positions minus 16000. Size of the nodes is proportional to the number of sequences included. Only selected mutations are shown. [file 1471-2148-9-63-S3.jpeg]
