# Supplementary material for: Migration of Chadic speaking pastoralists within Africa based on population structure of Chad Basin and phylogeography of mitochondrial L3f haplogroup
Source: BMC Evol Biol. 2009 Mar 23;9:63. doi: 10.1186/1471-2148-9-63 (PMC2680838; doi:10.1186/1471-2148-9-63)
Supplement: Additional file 4 — Matrix of FST between populations. Matrix of FST values derived from mtDNA HVS I sequences in African populations speaking Afro-Asiatic languages and the non-Afro-Asiatic speaking populations of Chad Basin; FST values below diagonal and significant values (p < 0.001) above diagonal. [file 1471-2148-9-63-S4.pdf]

Matrix of FSTs between populations and their significance levels (p = 0,001)

	Egyptians Lower	Egyptians Upper 1	Egyptians Upper 2	al-Hayez	Amhara 1	Amhara 2	Gurage	Saharawi	Maroccan Arab	Zriba	Arabs Chad	Arabs Shuwa	Tigrais	Oromo	Afars	Burunge	Iraqw	Somali	Hide	Kotoko	Mafa	Masa	Buduma	Hausa	Daba	Mandara	Ouldeme	Podokwo	Tuareg	Maroccan Berber	Berber Souss	Kesra	Fali2	Fulani Bongor	Fulani Tcheboua	Fulani1	Fali1	Fulani2	Tupuri	Yoruba	Kanembu	Kanuri	Kanuri W	Songhai
Egyptians Lower		-	-	-	+	+	-	+	-	+	-	+	-	-	-	+	+	+	+	+	+	+	+	+	+	+	+	+	+	+	-	-	+	+	+	+	+	+	+	+	+	+	-	-
Egyptians Upper 1	0,00		-	-	-	+	-	-	-	+	-	+	-	-	-	+	+	-	+	+	+	+	+	+	+	+	+	+	+	+	-	-	+	+	+	+	+	+	+	+	+	+	-	-
Egyptians Upper 2	0,02	0,02		-	-	-	-	+	-	+	-	+	-	-	-	+	+	-	-	+	+	+	+	+	+	+	-	+	+	+	+	-	+	+	+	+	+	+	-	+	+	-	-	-
al-Hayez	0,03	0,04	0,05		+	+	-	+	-	+	-	+	+	-	-	+	+	-	-	+	+	+	+	+	-	+	-	+	+	+	+	+	+	+	+	+	+	+	+	+	+	+	-	-
Amhara 1	0,02	0,03	0,02	0,05		-	-	+	-	+	-	+	-	-	-	+	+	-	-	+	+	+	+	+	-	+	-	+	-	+	+	+	+	+	+	+	+	+	-	-	+	-	-	-
Amhara 2	0,03	0,04	0,01	0,05	0,01		-	+	+	+	-	-	-	-	-	+	+	-	-	+	-	-	+	-	-	+	-	+	-	+	+	+	+	+	+	+	+	+	-	-	+	-	-	-
Gurage	0,04	0,04	0,02	0,07	0,00	0,00		+	-	+	-	-	-	-	-	+	+	-	-	-	-	-	-	-	-	-	-	-	-	+	+	-	-	+	-	-	-	-	-	-	-	-	-	-
Saharawi	0,05	0,04	0,06	0,14	0,09	0,11	0,13		-	-	+	+	+	+	+	+	+	+	+	+	+	+	+	+	+	+	+	+	+	-	-	+	+	+	+	+	+	+	+	+	+	+	+	+
Maroccan Arab	0,01	0,00	0,03	0,06	0,04	0,05	0,04	0,03		+	+	+	+	+	-	+	+	+	+	+	+	+	+	+	+	+	+	+	+	-	-	-	+	+	+	+	+	+	+	+	+	+	-	+
Zriba	0,05	0,05	0,08	0,15	0,10	0,11	0,15	0,03	0,05		+	+	+	+	+	+	+	+	+	+	+	+	+	+	+	+	+	+	+	+	+	+	+	+	+	+	+	+	+	+	+	+	+	+
Arabs Chad	0,03	0,04	0,03	0,02	0,02	0,01	0,03	0,17	0,08	0,16		-	-	-	-	+	+	-	-	-	-	-	-	-	-	-	-	-	-	+	+	-	-	-	-	-	-	-	-	-	-	-	-	-
Arabs Shuwa	0,07	0,08	0,05	0,09	0,04	0,02	0,04	0,20	0,10	0,20	0,01		+	-	-	+	+	-	-	-	-	-	-	-	-	+	-	-	-	+	+	-	-	+	+	-	+	-	-	-	+	-	-	-
Tigrais	0,01	0,02	0,01	0,06	0,00	0,00	0,00	0,08	0,04	0,09	0,03	0,05		-	-	+	+	-	+	+	+	+	+	+	+	+	+	+	-	+	+	+	+	+	+	+	+	+	+	+	+	+	-	-
Oromo	0,03	0,03	0,01	0,05	0,00	0,00	0,00	0,11	0,05	0,13	0,01	0,03	0,00		-	+	+	-	-	+	+	-	-	-	-	+	-	+	-	+	+	-	-	+	+	+	+	-	-	-	+	-	-	-
Afars	0,04	0,05	0,03	0,06	0,00	0,00	0,00	0,18	0,07	0,22	0,00	0,03	0,01	0,00		-	-	-	-	-	-	-	-	-	-	-	-	-	-	+	+	-	-	-	-	-	-	-	-	-	-	-	-	-
Burunge	0,25	0,26	0,17	0,22	0,15	0,14	0,14	0,38	0,25	0,45	0,18	0,21	0,18	0,14	0,11		-	+	+	+	+	+	+	+	+	+	+	+	+	+	+	+	+	+	+	+	+	+	+	+	+	+	+	+
Iraqw	0,29	0,31	0,19	0,24	0,17	0,16	0,17	0,53	0,34	0,66	0,23	0,31	0,20	0,16	0,14	0,02		+	+	+	+	+	+	+	+	+	+	+	+	+	+	+	+	+	+	+	-	+	+	+	+	+	+	-
Somali	0,04	0,04	0,03	0,07	0,01	0,00	0,00	0,16	0,06	0,16	0,02	0,01	0,01	0,01	0,01	0,17	0,23		-	-	-	-	-	-	-	-	-	-	-	+	+	-	-	+	+	-	-	-	-	-	-	-	-	-
Hide	0,06	0,08	0,05	0,06	0,03	0,02	0,02	0,20	0,09	0,22	0,00	0,01	0,05	0,03	0,00	0,15	0,18	0,01		-	-	-	-	-	-	-	-	-	-	+	+	-	-	-	-	-	-	-	-	-	-	-	-	-
Kotoko	0,10	0,11	0,06	0,12	0,05	0,04	0,05	0,20	0,12	0,21	0,05	0,03	0,07	0,05	0,04	0,19	0,24	0,03	0,01		-	-	-	-	-	+	-	-	-	+	+	+	-	+	+	+	+	+	-	-	+	-	-	-
Mafa	0,10	0,11	0,06	0,11	0,05	0,04	0,05	0,21	0,12	0,24	0,04	0,02	0,07	0,05	0,04	0,16	0,22	0,04	0,01	0,03		-	-	-	-	+	-	-	-	+	+	+	-	+	-	-	-	-	-	-	+	-	-	-
Masa	0,10	0,12	0,07	0,10	0,04	0,03	0,03	0,25	0,13	0,26	0,02	0,02	0,06	0,03	0,02	0,16	0,21	0,02	0,00	0,00	0,02		-	-	-	-	-	-	-	+	+	+	-	+	-	-	-	-	-	-	-	-	-	-
Buduma	0,08	0,09	0,07	0,10	0,05	0,04	0,04	0,21	0,11	0,20	0,03	0,03	0,06	0,04	0,04	0,22	0,29	0,02	0,00	0,03	0,04	0,03		-	-	-	-	-	-	+	+	+	-	+	+	-	-	-	-	-	-	-	-	-
Hausa	0,10	0,12	0,10	0,09	0,05	0,04	0,05	0,31	0,14	0,28	0,02	0,03	0,08	0,05	0,02	0,22	0,34	0,03	0,00	0,06	0,06	0,03	0,02		-	-	-	-	-	+	+	+	-	-	-	-	-	-	-	-	-	-	-	-
Daba	0,10	0,10	0,06	0,06	0,03	0,03	0,03	0,26	0,12	0,28	0,00	0,01	0,07	0,03	0,01	0,13	0,17	0,02	0,00	0,04	0,03	0,00	0,03	0,00		-	-	-	-	+	+	+	-	-	-	-	-	-	-	-	-	-	-	-
Mandara	0,10	0,12	0,09	0,08	0,04	0,05	0,04	0,27	0,12	0,27	0,03	0,04	0,08	0,05	0,01	0,19	0,26	0,03	0,00	0,06	0,06	0,02	0,03	0,00	0,00		-	-	-	+	+	+	-	-	-	-	-	-	-	-	-	-	-	-
Ouldeme	0,06	0,07	0,05	0,06	0,02	0,02	0,02	0,17	0,07	0,18	0,00	0,01	0,05	0,02	0,00	0,15	0,20	0,02	0,00	0,03	0,01	0,01	0,02	0,01	0,00	0,01		-	-	+	+	-	-	-	-	-	-	-	-	-	-	-	-	-
Podokwo	0,10	0,10	0,06	0,08	0,04	0,04	0,04	0,20	0,11	0,21	0,02	0,01	0,07	0,04	0,03	0,16	0,20	0,03	0,02	0,03	0,04	0,02	0,03	0,04	0,00	0,04	0,01		-	+	+	+	-	+	-	-	-	-	-	-	+	-	-	-
Tuareg	0,06	0,08	0,06	0,06	0,03	0,03	0,03	0,20	0,08	0,20	0,01	0,01	0,05	0,03	0,01	0,19	0,27	0,01	0,00	0,02	0,03	0,01	0,01	0,00	0,01	0,00	0,00	0,03		+	+	-	-	-	-	-	-	-	-	-	-	-	-	-
Maroccan Berber	0,06	0,05	0,07	0,14	0,08	0,11	0,11	0,01	0,00	0,05	0,16	0,18	0,08	0,11	0,17	0,37	0,48	0,14	0,19	0,19	0,21	0,23	0,20	0,25	0,23	0,23	0,15	0,19	0,17		+	+	+	+	+	+	+	+	+	+	+	+	+	+
Berber Souss	0,03	0,03	0,07	0,13	0,07	0,09	0,12	0,03	0,03	0,04	0,11	0,13	0,08	0,10	0,14	0,41	0,60	0,11	0,14	0,15	0,15	0,19	0,13	0,18	0,20	0,19	0,10	0,16	0,12	0,06		-	+	+	+	+	+	+	+	+	+	+	+	+
Kesra	0,02	0,02	0,03	0,06	0,03	0,03	0,04	0,07	0,03	0,07	0,03	0,04	0,03	0,03	0,04	0,26	0,34	0,04	0,05	0,06	0,08	0,07	0,05	0,07	0,08	0,07	0,02	0,06	0,03	0,07	0,03		+	+	+	+	+	+	-	-	+	+	-	-
Fali 2	0,10	0,11	0,07	0,10	0,04	0,04	0,04	0,24	0,13	0,26	0,03	0,02	0,07	0,03	0,01	0,14	0,17	0,03	0,00	0,02	0,03	0,00	0,04	0,03	0,00	0,02	0,00	0,02	0,02	0,22	0,18	0,07		+	-	-	-	-	-	-	-	-	-	-
Fulani Bongor	0,15	0,16	0,16	0,16	0,08	0,09	0,09	0,33	0,19	0,34	0,10	0,11	0,13	0,09	0,03	0,23	0,33	0,11	0,07	0,14	0,12	0,09	0,12	0,05	0,06	0,05	0,05	0,09	0,07	0,31	0,22	0,13	0,06		-	-	+	-	+	-	+	+	-	-
Fulani Tcheboua	0,12	0,13	0,12	0,14	0,05	0,06	0,05	0,29	0,16	0,30	0,06	0,05	0,09	0,06	0,01	0,22	0,30	0,07	0,03	0,09	0,05	0,04	0,06	0,03	0,02	0,02	0,02	0,05	0,04	0,27	0,18	0,10	0,03	0,00		-	-	-	-	-	-	-	-	-
Fulani 1	0,08	0,10	0,08	0,10	0,04	0,04	0,04	0,20	0,10	0,20	0,03	0,03	0,07	0,04	0,01	0,22	0,31	0,04	0,02	0,06	0,05	0,03	0,04	0,00	0,01	0,02	0,00	0,03	0,01	0,19	0,12	0,05	0,02	0,02	0,01		+	-	-	-	-	-	-	-
Fali1	0,13	0,14	0,09	0,10	0,05	0,05	0,05	0,30	0,16	0,30	0,03	0,05	0,09	0,05	0,02	0,14	0,17	0,05	0,01	0,06	0,05	0,02	0,04	0,02	0,00	0,01	0,02	0,04	0,02	0,27	0,24	0,12	0,02	0,09	0,05	0,05		-	-	-	-	-	-	-
Fulani 2	0,07	0,09	0,07	0,07	0,03	0,04	0,05	0,20	0,10	0,21	0,02	0,02	0,07	0,04	0,01	0,20	0,29	0,04	0,02	0,06	0,04	0,04	0,04	0,01	0,01	0,01	0,00	0,03	0,01	0,19	0,11	0,05	0,02	0,03	0,02	0,00	0,04		-	-	-	-	-	-
Tupuri	0,07	0,09	0,04	0,10	0,02	0,02	0,03	0,18	0,09	0,20	0,03	0,01	0,05	0,02	0,01	0,16	0,21	0,02	0,01	0,01	0,01	0,01	0,03	0,05	0,02	0,04	0,00	0,02	0,01	0,17	0,13	0,04	0,01	0,10	0,05	0,03	0,05	0,03		-	-	-	-	-
Yoruba	0,07	0,10	0,06	0,07	0,03	0,03	0,03	0,23	0,10	0,25	0,02	0,03	0,06	0,03	0,00	0,14	0,21	0,03	0,00	0,04	0,03	0,02	0,03	0,00	0,00	0,00	0,00	0,03	0,00	0,21	0,15	0,05	0,00	0,03	0,01	0,00	0,02	0,00	0,01		-	-	-	-
Kanembu	0,10	0,12	0,09	0,09	0,05	0,04	0,04	0,25	0,13	0,26	0,02	0,04	0,08	0,05	0,00	0,16	0,21	0,03	0,00	0,04	0,06	0,02	0,03	0,01	0,02	0,00	0,01	0,04	0,00	0,24	0,18	0,07	0,02	0,05	0,03	0,03	0,02	0,03	0,04	0,00		-	-	-
Kanuri	0,08	0,09	0,05	0,10	0,03	0,03	0,03	0,18	0,10	0,20	0,02	0,00	0,05	0,03	0,02	0,18	0,23	0,02	0,00	0,00	0,00	0,00	0,01	0,02	0,01	0,04	0,00	0,01	0,01	0,17	0,12	0,05	0,01	0,08	0,03	0,01	0,03	0,02	0,00	0,02	0,03		-	-
Kanuri W	0,06	0,07	0,05	0,06	0,02	0,02	0,02	0,22	0,08	0,23	0,00	0,00	0,05	0,02	0,00	0,16	0,23	0,01	0,00	0,01	0,02	0,00	0,00	0,00	0,00	0,00	0,00	0,00	0,00	0,18	0,14	0,03	0,00	0,06	0,02	0,00	0,02	0,00	0,00	0,00	0,00	0,00		-
Songhai	0,08	0,10	0,10	0,05	0,05	0,06	0,05	0,26	0,11	0,29	0,02	0,07	0,08	0,05	0,01	0,16	0,20	0,06	0,01	0,11	0,06	0,05	0,05	0,01	0,00	0,02	0,00	0,05	0,01	0,22	0,18	0,09	0,04	0,04	0,02	0,02	0,03	0,02	0,08	0,01	0,02	0,06	0,01	

Linguistic groupings:

- Semitic
- Cushitic
- Chadic
- Berber
- Non-Afro-Asiatic
